# Bovine Respiratory Mycoplasmas and the Commensal–Pathogen Continuum: A Systematic Review of Vaccines and Diagnostic Approaches

**DOI:** 10.3390/ani16060960

**Published:** 2026-03-19

**Authors:** Gebremeskel Mamu Werid, Yassein M. Ibrahim, Ashenafi Kiros Wubshet, Joshua W. Aleri, Farhid Hemmatzadeh, Kiro R. Petrovski

**Affiliations:** 1 Australian Centre for Antimicrobial Resistance Ecology, School of Animal and Veterinary Sciences, Adelaide University, Roseworthy, SA 5371, Australiafarhid.hemmatzadeh@adelaide.edu.au (F.H.); 2 Davies Livestock Research Centre, School of Animal and Veterinary Sciences, Adelaide University, Roseworthy, SA 5371, Australia; 3 Chongqing Academy of Animal Science, Chongqing 402460, China; yassin8322@gmail.com; 4 National Centre of Technology Innovation for Pigs, Chongqing 402460, China; 5 Faculty of Veterinary Science, University of Nyala, Nyala 155, Sudan; 6 Shandong Binzhou Institute of Animal Husbandry and Veterinary Sciences, Binzhou 256600, China; nafikw@gmail.com; 7 College of Veterinary Medicine, Shandong Agricultural University, Taian 271018, China; 8 School of Veterinary Science, The University of Queensland, Gatton, QLD 4343, Australia; j.aleri@uq.edu.au

**Keywords:** carriage, diagnostic test accuracy, immunisation, pathobiont

## Abstract

Mycoplasmas colonise bovine respiratory mucosal surfaces as commensal organisms, yet certain species may contribute to bovine respiratory disease complex (BRDC) when host and environmental conditions favour pathogenic expression. Clinical outcome is context-dependent, with species ranging from assumed true commensals (*M. arginini*, *M. bovirhinis*) to pathobionts (*M. bovis*) and less frequently reported species (*M. alkalescens*, *M. canadense*) and an opportunist (*M. dispar*). The absence of a synthesis applying a commensal–pathogen continuum framework to bovine respiratory mycoplasmas while jointly evaluating carriage, vaccine performance, and diagnostic interpretability represents a key gap. The objective of this paper is to evaluate available evidence on vaccination, diagnostics, and control of bovine respiratory mycoplasmas within a commensal–pathogen continuum framework. The preparation of this paper followed the preferred reporting items for systematic reviews and meta-analyses (PRISMA) 2020 and synthesis without meta-analysis (SWiM) guidelines. PubMed, Scopus, and Web of Science were searched through 12 December 2025. Of 6119 records identified, 212 studies met predefined Population, Intervention, Comparator, Outcome, Study design (PICOS) criteria and were classified into four domains: carriage and prevalence (*n* = 73), diagnostic performance (*n* = 71), pathogenesis and immune evasion (*n* = 53), and vaccine efficacy (*n* = 15). Risk of bias was assessed using domain-appropriate tools. Evidence certainty was evaluated using the Grading of Recommendations, Assessment, Development and Evaluation (GRADE) framework. *M. bovis* dominated the literature (199/212; 93.9%), reflecting concentrated research investment, with *M. dispar* (22; 10.4%), *M. bovirhinis* (19; 9.0%), *M. arginini* (4; 1.9%), *M. canadense* (1; 0.5%), and *M. alkalescens* (1; 0.5%) also well documented. *M. bovirhinis* and *M. arginini* were consistently recovered from clinically healthy cattle, supporting their classification as true commensals. *M. bovis* exhibited pathobiont behaviour. Nasopharyngeal carriage was reported in 18–58% of healthy cattle and progressed to clinical disease (estimated 15–40%) in a context-dependent manner. Whole-cell bacterins demonstrated inconsistent efficacy, whereas virulence-factor vaccines showed more consistently positive outcomes. Future vaccines targeting conserved virulence-associated antigens and designed to elicit mucosal immunity may provide higher levels and more consistent protection than conventional whole-cell bacterin formulations. The majority of diagnostic studies detected mycoplasma presence without distinguishing colonisation from causation. Bovine respiratory mycoplasma species occupy distinct positions on the commensal–pathogen continuum, with direct implications for vaccine design, diagnostic interpretation, and disease control. Integrated control combining syndrome-aligned diagnostics and targeted vaccination was the approach most consistently supported by the available evidence.

## Simple Summary

*Mycoplasmas* commonly colonise the respiratory tract of healthy cattle, yet under stress, viral co-infection, or weakened immunity, some species contribute to serious respiratory disease. Species differ in pathogenic potential: *Mycoplasma bovirhinis* and *Mycoplasma arginini* are assumed to be harmless commensals, whereas *Mycoplasma bovis* can shift from silent colonisation to active disease depending on host and environmental conditions. This systematic review evaluated vaccines and diagnostics for bovine respiratory mycoplasmas within a commensal–pathogen continuum framework. Conventional killed vaccines showed inconsistent efficacy, while newer formulations targeting specific virulence factors demonstrated more consistent protection. Critically, detecting mycoplasmas alone did not distinguish harmless carriage from disease causation. Effective control requires diagnostics that differentiate carriage from infection, vaccines that prevent disease rather than eliminate colonisation, and integrated biosecurity practices.

## 1. Introduction

Bovine respiratory mycoplasmas are important contributors to bovine respiratory disease (BRD; also referred to as bovine respiratory disease complex [BRDC]) and are responsible for significant production losses, animal welfare impacts, and increased antimicrobial use globally [1,2,3]. The significance of mycoplasmas is amplified by their persistent endemicity in most cattle production systems and by the capacity of several species to colonise clinically healthy cattle, complicating diagnostic interpretation and disease attribution.

A defining feature of bovine respiratory mycoplasmas is their position on a commensal–pathogen continuum, whereby clinical outcome is context-dependent rather than determined solely by organism detection [4,5]. Not all species behave identically. *Mycoplasma bovirhinis* demonstrates near-universal nasopharyngeal carriage in healthy cattle yet is rarely associated with clinical disease, representing the commensal end of the spectrum [6,7]. *M. arginini*, a multi-host commensal frequently co-isolated from bovine respiratory samples, similarly occupies a non-pathogenic position [8]. By contrast, *M. bovis* is best characterised as a pathobiont: reported nasopharyngeal carriage prevalence is substantial in clinically healthy cattle [9,10,11,12], yet only a minority of colonised cattle develop clinical disease, with progression associated with viral co-infection, transport stress, and young age [13,14,15,16]. *M. dispar* occupies an intermediate position as a commensal or opportunist frequently isolated from the bovine respiratory tract that may contribute to the BRDC as a co-pathogen [17,18]. *M. alkalescens* and *M. canadense* are occasionally recovered from respiratory specimens, though their pathogenic roles in BRDC remain uncertain [3]. Accordingly, detection of a bovine respiratory mycoplasma should not be interpreted as equivalent to disease causation [4,5]; rather, interpretation requires a species-aware framework that integrates ecology, host context, and diagnostic setting.

The damage–response framework [4] provides theoretical grounding for these observations: detection reflects colonisation, whereas disease reflects the integrated effect of microbial virulence, host immune status, and environmental stressors. Reduced genomes of mycoplasmas (0.580–1.35 Mb) [19,20] may reflect adaptation mechanisms to host dependence and metabolic streamlining rather than a direct mechanism of persistence. By contrast, the absence of a cell wall contributes to immune evasion and altered susceptibility to host defence mechanisms, while biofilm formation [21], antigenic variation, and immunomodulation are more directly implicated in stable mucosal persistence. Bovine respiratory disease complex (BRDC) has been linked to annual losses exceeding USD 900 million in North America [1,2], and *M. bovis* is increasingly recognised as a key contributor to this polymicrobial syndrome [22,23].

Control of mycoplasma-associated BRDC remains challenging because biological mechanisms, including high-frequency antigenic variation through variable surface proteins (Vsps) [24,25], phase switching [26], active immunomodulation [27,28], and biofilm-associated tolerance [21], promote persistence and immune evasion. Vaccination has largely relied on inactivated whole-cell formulations with inconsistent efficacy [29,30,31], and correlations of protection remain incompletely defined. Although molecular assays have improved analytical sensitivity [32,33], high background carriage in the upper respiratory tract increases interpretive uncertainty unless results are integrated with clinical syndrome, quantitative burden, tissue localisation, and epidemiological context. Sampling depth further affects inference, with deep nasopharyngeal sampling showing higher agreement with bronchoalveolar lavage than superficial nasal swabs [34,35].

Existing reviews of *M. bovis* have typically focused on molecular epidemiology, virulence mechanisms, or antimicrobial resistance in isolation, without integrating carriage ecology, diagnostic interpretability, or species-level variation along a commensal–pathogen gradient [1,2,3]. A key research gap is therefore the absence of a consolidated synthesis that explicitly applies a commensal–pathogen continuum framework to bovine respiratory mycoplasmas while jointly evaluating carriage prevalence, species-specific pathogenic potential, vaccine performance, and diagnostic interpretability. The current evidence base is fragmented across species, study designs, and endpoints, limiting comparability and complicating the determination of whether detection reflects clinically relevant infection or incidental colonisation. Addressing this gap is important because species misclassification can drive inappropriate antimicrobial use, reduce diagnostic specificity, and misdirect prevention resources.

This systematic review evaluated available evidence on vaccination, diagnostics, and control of bovine respiratory mycoplasmas within a commensal–pathogen continuum by characterising where individual mycoplasma species sit on the continuum, synthesising carriage prevalence in clinically healthy cattle, comparing vaccine efficacy across platforms and delivery routes, and identifying diagnostic approaches and integrated control strategies most consistently supported by the available evidence.

## 2. Materials and Methods

### 2.1. Protocol and Reporting Guidelines

This systematic review with narrative synthesis was conducted in accordance with the preferred reporting items for systematic reviews and meta-analyses (PRISMA) 2020 guidelines [36] and the synthesis without meta-analysis (SWiM) reporting guideline [37]. The PRISMA checklist is provided in the Supplementary Material S1 and the SWiM checklist in Supplementary Material S6. No deviations from the registered protocol occurred.

### 2.2. Eligibility Criteria

Eligibility criteria were defined using the Population, Intervention/Exposure, Comparator, Outcome, and Study design (PICOS) framework. Inclusion and exclusion criteria are presented in Table 1. Studies were eligible if they addressed mycoplasma infection, carriage, vaccination, or diagnostics in domestic cattle (*Bos taurus*, *Bos indicus*, and their crosses) within the context of bovine respiratory disease. Studies exclusively addressing non-bovine species were excluded unless findings were directly applicable to domestic cattle respiratory disease. Exclusion criteria were applied hierarchically; any single exclusion criterion was sufficient to exclude a record. Records meeting no exclusion criteria but with indeterminate inclusion status were classified as ‘Uncertain’ and advanced to full-text assessment. Criterion E8 (vaccine design) was applied at the full-text stage, as randomisation details are frequently absent from abstracts in veterinary vaccine literature.

The target mycoplasma species reflected the commensal–pathogen continuum within the bovine respiratory tract. Six species repeatedly reported in three or more independent publications as isolated or detected from the bovine respiratory tract were within scope: *M. alkalescens* (*Metamycoplasma alkalescens*; occasional respiratory isolate), *M. arginini* (*Mycoplasmopsis arginini*; true commensal), *M. bovis* (*Mycoplasmopsis bovis*; pathobiont), *M. bovirhinis* (*Mycoplasmopsis bovirhinis*; true commensal), *M. canadense* (*Metamycoplasma canadense*; occasional respiratory isolate), and *M. dispar* (*Mesomycoplasma dispar*; commensal/opportunist). Revised generic names follow Gupta et al. [38] and the List of Prokaryotic Names with Standing in Nomenclature [39]. Studies addressing multiple mycoplasma species were included regardless of which species were identified, provided at least one mycoplasma species was reported within a bovine respiratory context. Studies focused exclusively on contagious bovine pleuropneumonia (CBPP) or contagious caprine pleuropneumonia (CCPP) were outside the scope of this review (exclusion criterion E6) and are referenced only for contextual comparison where explicitly labelled as out-of-scope [40,41].

### 2.3. Information Sources and Search Strategy

A comprehensive literature search was conducted across three electronic databases: PubMed/MEDLINE, Web of Science Core Collection, and Scopus. The search strategy was developed iteratively with input from a research librarian (acknowledged) to capture literature indexed under both legacy (*Mycoplasma*) and revised nomenclature (*Mycoplasmopsis, Mesomycoplasma,* and *Metamycoplasma*), reflecting taxonomic revisions validated under the International Code of Nomenclature of Prokaryotes [38,39]. The search combined three concept blocks using Boolean operators (Table 2). The final search was executed on 12 December 2025 at 05:00 Australian Central Daylight Time (ACDT; UTC+10:30). To ensure currency of the evidence base, the search was updated during manuscript revision to capture newly indexed and early online records published until 9 February 2026; records identified during this update were screened and assessed using the same predefined eligibility criteria. Complete search strategies with all Boolean operators and field tags are provided in Supplementary Material S2.

### 2.4. Study Selection

Records were exported to EndNote X21 (Clarivate Analytics, Philadelphia, PA, USA) and duplicates removed using the “find duplicates” function with conservative matching settings, followed by manual verification. Deduplicated records were imported into Covidence systematic review software (https://app.covidence.org) for screening.

Title and abstract screening were conducted independently by two reviewers (GMW, YMI) against predefined eligibility criteria using Covidence’s blinded screening function, following a hierarchical decision. Exclusion criteria (E1–E7) were assessed first; any single criterion was sufficient to exclude a record. Records meeting no exclusion criteria were assessed against the inclusion criteria. Records satisfying all inclusion criteria were classified as ‘Include’; records with indeterminate status advanced to full-text assessment, consistent with Cochrane guidance to favour inclusion when in doubt at the title and abstract stage.

Full-text articles of potentially eligible records were retrieved and assessed independently by both reviewers. Exclusion criterion E8 (vaccine study design) was applied at this stage. Disagreements were resolved by consensus discussion or, where consensus could not be reached, by consultation with a third reviewer (AKW). Reasons for exclusion at the full-text stage were documented in Covidence and are reported in the PRISMA flow diagram (Supplementary Figure S1). Inter-rater reliability was assessed using Cohen’s kappa (κ): κ = 0.780 (95% confidence interval [CI]: 0.740–0.820) at title and abstract screening and κ = 0.910 (95% CI: 0.870–0.950) at full-text assessment, indicating substantial to almost perfect agreement [42].

### 2.5. Data Extraction

Data were extracted using a standardised template developed in Microsoft Excel (Microsoft Corporation, Redmond, WA, USA) and piloted on 20 randomly selected studies. Pilot extraction was performed independently by two reviewers (GMW, YMI), with discrepancies resolved through template refinement. Following piloting, data were extracted by one reviewer and verified by a second (duplicate extraction on 15% of studies; discrepancy rate <5%). The extraction template (Supplementary Material S3) captured variables across eight domains: study identification, design, population, clinical context, target organism(s) and continuum classification, co-pathogen assessment, vaccine details, and diagnostic details. 

### 2.6. Risk of Bias Assessment

Risk of bias was assessed independently by two reviewers (GMW, AKW), with disagreements resolved by discussion. Design-appropriate tools were applied: the Cochrane Risk of Bias tool version 2 (RoB 2) for randomised trials [43]; risk of bias in non-randomised studies of interventions (ROBINS-I) for non-randomised intervention studies [44]; quality assessment of diagnostic accuracy studies version 2 (QUADAS-2) for diagnostic accuracy studies [45]; and the Newcastle–Ottawa Scale (NOS) for observational studies [46]. Quality ratings informed the weighting of evidence in narrative synthesis and GRADE assessments, but were not used to exclude studies. Each of the assessments are provided in Supplementary Material S5. Risk-of-bias assessments were used to inform the weighting and interpretation of evidence within each domain. Studies judged to be at high risk of bias were assigned less weight in the narrative synthesis, and domain-level GRADE certainty ratings [47] were downgraded where risk of bias, inconsistency, or indirectness was considered significant (Supplementary Material S4).

### 2.7. Synthesis Methods

Quantitative meta-analysis was precluded by substantial clinical and methodological heterogeneity. Evidence was synthesised using thematic narrative synthesis following SWiM guidelines [37]. Studies were grouped into predefined thematic domains: (i) commensal carriage and species-specific continuum classification; (ii) vaccine efficacy; (iii) diagnostic performance; and (iv) pathogenesis and immune evasion. Effect-direction analysis summarised findings within each domain, with effect direction classified as positive (+, statistically significant benefit), null (○, no significant effect), or negative (−, statistically significant harm). Effect sizes with 95% confidence intervals were reported where available. Effect-direction analysis constitutes a structured form of vote counting that summarises the direction and statistical significance of individual study findings. It does not estimate pooled effect magnitude, and statistical significance thresholds, models, and outcome definitions varied across included studies. Formal assessment of publication bias through funnel plots or statistical tests was not planned because the heterogeneity of study designs, outcomes, and species across domains precluded the calculation of comparable effect estimates required for such assessment. Due to higher heterogeneity in sampling site, disease spectrum, target species, index test design, threshold definition, and reference standard, pooled sensitivity and specificity estimates and direct cross-study comparisons of diagnostic accuracy were not undertaken.

### 2.8. Certainty of Evidence Assessment

Certainty of evidence was assessed using the Grading of Recommendations, Assessment, Development and Evaluation (GRADE) approach [47]. GRADE Summary of Findings tables were constructed for key comparisons. For diagnostic accuracy studies, GRADE was adapted to assess certainty in estimates of sensitivity (Se) and specificity (Sp), with particular attention to spectrum bias, reference standard validity, and applicability. Where diagnostic accuracy evidence was synthesised narratively, interpretation emphasised applicability, bias, and reference-standard variability rather than pooled summary measures.

## 3. Results

### 3.1. Study Selection and Screening

The systematic search identified 6119 records: PubMed (*n* = 1562), Scopus (*n* = 2461), and Web of Science (*n* = 2096). Following duplicate removal (*n* = 2697), 3422 unique records underwent title and abstract screening. Of these, 2575 were excluded, leaving 847 full-text articles for eligibility assessment. Full-text review excluded 635 articles: wrong population (*n* = 201), reviews or meta-analyses (*n* = 112), insufficient methodological detail (*n* = 108), antimicrobial susceptibility without clinical context (*n* = 67), duplicate data (n = 54), methodology development without validation (*n* = 38), non-English language (*n* = 32), and other reasons (*n* = 23). A total of 212 studies met the inclusion criteria.

### 3.2. Characteristics of Included Studies

The 212 included studies were published between 1977 and 2026. Temporal distribution indicated accelerating research activity: 23 (10.8%) were published before 2000, 19 (9.0%) during 2000–2009, 88 (41.5%) during 2010–2019, and 82 (38.7%) during 2020–2026. Studies published until 09/02/2026 were included as part of the updated search conducted during manuscript revision. (Table 3). Studies were classified into four thematic domains: carriage and prevalence (73; 34.4%), diagnostic performance (71; 33.5%), pathogenesis and immune evasion (53; 25.0%), and vaccine efficacy (15; 7.1%).

Geographically, included studies originated from at least 30 countries across six continents (Table 3). Europe contributed the largest share (72; 34.0%), led by Belgium (12), Denmark (10), and Poland (9). The Americas accounted for 62 studies (29.2%), predominantly from the United States (24) and Canada (14). Asia contributed 42 studies (19.8%), with China (21) and Japan (8) as major contributors. Oceania was represented by 15 studies (7.1%), principally from Australia (12) and New Zealand (3). Africa (8; 3.8%) and South America (13; 6.1%) contributed smaller proportions. This geographic distribution reflects the concentration of cattle industries and mycoplasma research infrastructure in high-income regions, with limited representation from Africa, the Middle East, and Southeast Asia. Complete bibliographic details for all 212 included studies are provided in [6,7,8,9,10,11,12,13,14,15,16,17,18,22,23,25,27,28,30,31,32,33,34,35,48,49,50,51,52,53,54,55,56,57,58,59,60,61,62,63,64,65,66,67,68,69,70,71,72,73,74,75,76,77,78,79,80,81,82,83,84,85,86,87,88,89,90,91,92,93,94,95,96,97,98,99,100,101,102,103,104,105,106,107,108,109,110,111,112,113,114,115,116,117,118,119,120,121,122,123,124,125,126,127,128,129,130,131,132,133,134,135,136,137,138,139,140,141,142,143,144,145,146,147,148,149,150,151,152,153,154,155,156,157,158,159,160,161,162,163,164,165,166,167,168,169,170,171,172,173,174,175,176,177,178,179,180,181,182,183,184,185,186,187,188,189,190,191,192,193,194,195,196,197,198,199,200,201,202,203,204,205,206,207,208,209,210,211,212,213,214,215,216,217,218,219,220,221,222,223,224,225,226,227,228,229,230,231,232,233,234,235].

### 3.3. Risk of Bias and Evidence Certainty

Risk of bias assessments are summarised in Table 4. Among the 71 diagnostic accuracy studies assessed using QUADAS-2 [45], the risk of bias was low for patient selection and index test conduct in the majority. However, applicability concerns were noted for reference standards in approximately one-third, primarily owing to variability in reference standard selection. Among the 15 vaccine studies, RoB 2 [43] assessment identified some concerns regarding blinding in five studies and a high risk of bias for outcome measurement in two studies. Carriage and prevalence studies assessed with NOS [46] demonstrated moderate methodological quality overall, with convenience sampling and unclear case definitions identified as common limitations. GRADE certainty ratings [47] ranged from Very Low to Moderate across evidence domains, reflecting the predominance of observational study designs.

### 3.4. Mycoplasma Species Distribution and Continuum Classification

*M. bovis* was the most frequently studied species (199/212; 93.9%), followed by *M. dispar* (22; 10.4%), *M. bovirhinis* (19; 9.0%), *M. arginini* (4; 1.9%), *M. canadense* (1; 0.5%), and *M. alkalescens* (1; 0.5%). Totals exceeded 212 because some studies addressed multiple species. The species distribution across study domains is presented in Table 5.

The evidence supported distinct continuum positions for individual species. *M. bovirhinis* was recovered from the nasopharynx of more than 90% of sampled cattle in studies employing broad-spectrum detection, irrespective of respiratory disease status [6,7]. No included study attributed clinical disease to *M. bovirhinis* as a sole pathogen, supporting classification as a true commensal. *M. arginini* was similarly identified in clinically healthy and diseased cattle without evidence for a causal respiratory role [8]. *M. dispar* was frequently co-isolated with *M. bovis* and other BRDC pathogens [17,18] and was associated with mild calf pneumonia in some reports, consistent with an opportunistic or commensal role. *M. canadense* has been detected sporadically in bovine respiratory surveys but is poorly characterised in terms of respiratory pathogenicity, with available evidence limited to incidental detection in multi-species surveys [3].

*M. bovis* demonstrated the strongest evidence for pathobiont behaviour. A high rate of asymptomatic carriage with context-dependent disease progression was confirmed. Longitudinal studies documented nasopharyngeal colonisation preceding clinical disease, with progression associated with viral co-infection (bovine viral diarrhoea virus [BVDV], bovine herpesvirus 1 [BHV-1]) [13,16], transport stress [14], and young age (<6 months) [15,82]. Recent microbiome-based investigations demonstrated that pathobiont abundance did not reliably distinguish cattle with BRDC from clinically healthy cattle in all production contexts [74], further supporting the continuum model.

### 3.5. Carriage Prevalence in Clinically Healthy Cattle

A total of 73 studies examined mycoplasma carriage, prevalence, and within-herd dynamics. For *M. bovis*, nasopharyngeal carriage in clinically healthy cattle was consistently documented, with reported prevalence ranging from approximately 18% to 58% depending on detection method and sampling strategy [9,10,11,12]. Bulk tank milk surveillance generally demonstrated lower herd-level detection rates [130,162,178]. Recognised commensal species (*M. bovirhinis*, and *M. arginini*) were recovered from more than 85% of sampled cattle in multiple studies employing broad-spectrum detection [7]. Longitudinal data indicated that among *M. bovis*-colonised cattle, only an estimated 15–40% developed clinical disease, with progression associated with viral co-infection (BVDV or BHV-1) [13,16], transport stress [14], and young age [15,82].

### 3.6. Diagnostic Test Performance

A total of 71 studies evaluated diagnostic test performance. The majority detected mycoplasma presence without tissue localisation evidence sufficient to distinguish active infection from commensal carriage [32,33,155,175]. Culture-based detection demonstrated high specificity but variable sensitivity (approximately 42–78%) compared with polymerase chain reaction (PCR) [32,33]. Quantitative PCR achieved high sensitivity and specificity for *M. bovis* [113,143,175]. Sampling technique significantly affected results: deep nasopharyngeal swabs showed approximately 87% agreement with bronchoalveolar lavage, compared with only 52% for superficial nasal swabs [34,35,213]. Loop-mediated isothermal amplification (LAMP) assays demonstrated promise for rapid, field-deployable detection [56,92,126,174]. Multiplex platforms enabled simultaneous detection of multiple respiratory pathogens, including co-detection of *M. bovis* with *M. dispar* and *M. bovirhinis* [23,155]. Matrix-assisted laser desorption/ionisation time-of-flight mass spectrometry (MALDI-TOF MS) [62] and nanopore sequencing [63] provided novel species- and strain-level identification. Serological methods detected immune responses rather than current infection [100,156]. Reported diagnostic performance varied across studies according to sampling site, disease definition, pathogen target, and reference standard.

### 3.7. Vaccine Efficacy by Platform

A total of 15 studies evaluated vaccine efficacy using controlled designs (Table 6). Whole-cell *M. bovis* bacterins demonstrated inconsistent efficacy: two randomised controlled trials reported no significant reduction in pneumonia incidence [30,31], with one documenting significantly increased otitis media in vaccinated calves [31]. Subunit vaccines targeting virulence factors (elongation factor Tu, heat shock protein 70) demonstrated partial protection in experimental challenge models [136]. Intranasal modified-live delivery showed promise for inducing mucosal immunity [67]. Combined *M. bovis*–BoHV-1 vaccine approaches demonstrated broad protection in recent experimental studies [109,232]. Saponin-based adjuvant formulations stimulated both T helper type 1 (Th1) and T helper type 2 (Th2) responses [88,90]. The only vaccine study addressing a species other than *M. bovis* evaluated a *M. dispar* component within a multivalent respiratory vaccine [129]. The observed vaccine efficacy varied across studies according to one or more of the factors, such as antigen formulation, adjuvant, route of delivery, challenge model, endpoint definition, and follow-up duration.

### 3.8. Pathogenesis and Immune Evasion

A total of 53 studies examined pathogenesis and immune evasion mechanisms, predominantly in *M. bovis* (47/53; 88.7%). Six principal biological features were identified that collectively undermine conventional vaccination and explain *M. bovis* pathobiont behaviour: (i) absence of a cell wall, eliminating classical pathogen-associated molecular patterns (PAMPs) [19,26]; (ii) high-frequency antigenic variation through Vsps [24,25]; (iii) intracellular survival within non-professional phagocytes [166,198]; (iv) active immunomodulation through arginine depletion and apoptosis modulation [27,28,78]; (v) biofilm-mediated persistence [21]; and (vi) mucosal immune compartmentalisation [67,111]. Mycoplasmal lipoproteins activated Toll-like receptor 2 (TLR2) signalling with Th2-biased responses [224], and chronic pneumonia models demonstrated persistent infection despite strong humoral responses [125,217]. Two studies examining *M. bovirhinis* identified limited tissue invasion and negligible inflammatory response compared with *M. bovis*, consistent with commensal behaviour. Novel virulence factors identified included fibronectin-binding adhesin P27 [77] and plasminogen-binding fructose-1,6-bisphosphate aldolase [101].

## 4. Discussion

This systematic review synthesised evidence from 212 original research studies on bovine respiratory mycoplasmas within a commensal–pathogen continuum framework. Bovine respiratory mycoplasma species occupied distinct, evidence-supported positions on this continuum, with direct implications for diagnosis, vaccination, and disease control [4,5]. Diagnostic interpretation was constrained by high background carriage and limited tissue-localisation evidence, reducing causal certainty when molecular detection was used alone [32,33,143]. Vaccine effects varied by platform and route, with mucosal delivery and targeted-antigen approaches showing more consistent lesion reduction than parenteral whole-cell bacterins [30,31,67,136], although field generalisability remained limited. Carriage was common and progression to clinical disease appeared context-dependent, with co-infection and management stressors recurring as plausible drivers [13,14,16], supporting integrated control strategies rather than single-test or single-intervention approaches [132,163]. These findings should be interpreted in the context of substantial study heterogeneity that precluded meta-analysis [37], concentration of evidence on *M. bovis* (93.9% of included studies), predominantly Low to Very Low GRADE certainty [47], and a small vaccine evidence base (*n* = 15 controlled studies).

The species-level analysis revealed differences with direct implications for diagnostic interpretation and disease attribution. *M. bovirhinis* was recovered from the nasopharynx in more than 90% of sampled cattle irrespective of respiratory disease status [6,7], and no included study attributed clinical disease to this species as a sole pathogen. *M. arginini* was similarly identified in both clinically healthy and diseased cattle without evidence for a causal respiratory role [8]. These observations, drawn from multi-species surveys employing both culture and molecular detection, consistently supported the classification of both species as true commensals. The practical consequence is that multiplex diagnostic panels reporting *M. bovirhinis* or *M. arginini* positivity without species-specific clinical interpretation risk unnecessary antimicrobial treatment [23,155].

*M. dispar* occupied a less clearly defined position. Although frequently co-isolated with *M. bovis* and other BRDC pathogens [17,18], attributing an independent pathogenic role was complicated by its high carriage rate in healthy calves and the polymicrobial context in which it was typically detected [22,23]. The available evidence was more consistent with a commensal or opportunistic role than with primary pathogenicity [3,17]. However, only four pathogenesis studies and one vaccine study addressed *M. dispar* directly, and none employed controlled challenge designs isolating its contribution from co-pathogens; the strength of this classification is therefore limited by the paucity of dedicated investigation. Therefore, a final characterisation of its pathogenicity was not attempted.

*M. bovis* provided the strongest evidence for pathobiont behaviour. Nasopharyngeal carriage was confirmed in 18–58% of clinically healthy cattle [9,10,11,12], with clinical disease developing in only a minority of colonised animals [13,14,15,16,82]. The immune evasion mechanisms identified for *M. bovis,* namely cell wall absence, high-frequency antigenic variation through variable surface proteins, intracellular survival within non-professional phagocytes, active immunomodulation, biofilm-mediated persistence, and mucosal immune compartmentalisation, may have enabled sustained asymptomatic colonisation while retaining the capacity for pathogenic expression under permissive host and environmental conditions. This combination of evasion strategies explained both the context-dependent progression from carriage to disease and the poor performance of conventional whole-cell bacterins, which elicit systemic humoral responses that are insufficient against an organism that varies its surface antigens, survives within host cells, and actively suppresses immune effector function [125,217]. In contrast, comparative pathogenesis data indicated that *M. bovirhinis* exhibited limited tissue invasion and negligible inflammatory response [55,194], which confirmed that these evasion mechanisms are species-specific rather than class-wide attributes of bovine Mollicutes and provided the biological basis for species-level differentiation along the commensal–pathogen continuum. The damage–response framework [4] provides theoretical grounding: detection reflects colonisation, whereas disease reflects the integrated effect of microbial virulence, host immune status, and environmental stressors, including viral co-infection (BVDV, BHV-1) [13,16], transport stress [14], and young age [15,82]. This strong evidence base for *M. bovis* contrasts with comparatively limited data for other species. The overwhelming concentration of included studies on a single species, while reflecting genuine economic importance [1,2,3], meant that continuum classifications for *M. dispar*, *M. bovirhinis*, and *M. arginini* relied partly on multi-species surveys rather than dedicated investigations.

The combination of frequent co-infection and the heavy research emphasis on *M. bovis* introduces a systematic inference bias that warrants explicit acknowledgement. Because bovine respiratory disease is typically polymicrobial [22,23], *M. bovis* is frequently co-detected with *M. dispar*, *M. bovirhinis*, and other bovine *Mollicutes*, yet the overwhelming majority of pathogenesis and vaccine studies have been designed and powered to detect *M. bovis* effects only. This asymmetry can bias inference in two directions. The pathogenic contribution of co-detected species, particularly *M. dispar*, for which only four pathogenesis studies and one vaccine study were available, may be systematically underestimated, because studies treating *M. bovis* as the primary or sole etiological agent seldom accounted for co-present *Mollicutes* as potential component causes [236]. Conversely, the role of *M. bovis* itself may be overestimated in settings where its detection coincides with co-infections by viral agents (BHV-1, BVDV, bovine respiratory syncytial virus [BRSV]) and bacterial pathogens (e.g., *Mannheimia haemolytica*, *Pasteurella multocida*, and *Histophilus somni*) that may independently cause or exacerbate BRDC lesions [13,16]. Attribution of disease to *M. bovis* in studies that did not employ multiplex diagnostics, quantitative load assessment, or tissue co-localisation techniques, therefore, remains confounded. This dual bias, simultaneous underestimation of the pathogenic contribution of other bovine *Mollicutes* and potential overestimation of the independent role of *M. bovis*, reinforces the need for multiplex diagnostic approaches that assess the full *Mollicute* community, quantitative burden, and tissue co-localisation within BRDC lesions [113,155,175]. Without such evidence, species-specific continuum positions remain provisional and are likely shaped as much by research investment patterns as by underlying biology.

The diagnostic evidence revealed a fundamental interpretive challenge. The positive detection of a mycoplasma species cannot, by itself, categorise cattle as ‘carrier’ or ‘diseased’. This is because bovine respiratory mycoplasmas exist in a state of co-presence within the nasopharyngeal microbiome, with true commensals (e.g., *M. bovirhinis* and *M. arginini*), opportunists (*M. dispar*), and a pathobiont (*M. bovis*) frequently co-inhabiting the same anatomical site [6,7,11]. Identical positive PCR results can represent benign commensal colonisation, pathobiont expansion during intercurrent illness, or primary pathogen replication causing tissue damage [5,74]. In BRDC, which is typically polymicrobial rather than mono-etiological [22,23], the concept of component causes versus sufficient causes [236] provides a useful framework. A sufficient cause comprises multiple components (for example, viral infection plus mycoplasma plus transport stress plus young age), none of which alone is sufficient. Accordingly, detection of any single mycoplasma species in this polymicrobial context supports association rather than sole causation, unless corroborated by multiplex tissue localisation, temporal sampling data, and concordant host-response markers [111,113,155].

The diagnostic significance of mycoplasma detection varied by species, specimen type, and production context. For true commensals such as *M. bovirhinis*, a positive result carried little diagnostic value for BRDC [7]. For *M. bovis*, detection alone was insufficient for causal attribution because the organism was also recovered from clinically healthy cattle [9,12]. Confidence in pathogenic involvement increased when supported by concordant evidence from several lines of investigation. Detection in lower respiratory tract specimens, including bronchoalveolar lavage, transtracheal wash, or deep nasopharyngeal swabs, provided stronger causal evidence than detection from superficial nasal swabs [34,35,213]. High organism burden expressed as standardised genome copies per microlitre or reaction volume rather than assay-specific cycle-threshold values further strengthened the association [143]. Additional supporting evidence included spatial localisation within lesions by immunohistochemistry [22], temporal association with disease onset through sequential sampling [11,14], and exclusion of alternative aetiological explanations through multiplex co-detection [113,155,175]. Host-response markers, including transcriptomic signatures, cytokine profiles, and acute-phase proteins, provided complementary but not yet definitive evidence in isolation [83,111]. These evidentiary elements formed an evidence ladder linking detection context to interpretive confidence, ranging from probable carriage to probable causal contribution. Within this framework, no universally validated quantitative PCR threshold distinguished asymptomatic carriage from active clinical infection. Klompmaker et al. [143] proposed a quantification cycle (Cq) cut-off of 21.3 or lower for *M. bovis* in a high-throughput real-time PCR system, but the predictive value of this threshold was population- and production-system-dependent. In beef feedlots, nasal pathobiont abundance was only a moderate indicator of BRD, and the strength of the association varied between feedlots [73]; in dairy cattle, commensal and pathobiont genera were prevalent regardless of disease status, and farm-level effects exceeded disease-status effects [74]. Francoz et al. [98] reported that *M. bovis* was the only pathogen assessed to be associated with higher odds of clinical signs, lung consolidation, and reduced average daily gain in preweaned dairy calves, and de la Fe et al. [81] found that respiratory signs appeared earlier in feedlot batches with higher initial *M. bovis* prevalence. These findings collectively indicated that quantitative burden should be interpreted in conjunction with specimen type, lesion context, co-detection profile, production setting, and clinical presentation.

Sampling frame and test modality influenced causal inference in ways that were inconsistently acknowledged in the included literature. Deep nasopharyngeal swabs showed approximately 87% agreement with bronchoalveolar lavage, compared with only 52% for superficial nasal swabs [35]. Therefore, studies relying exclusively on superficial sampling conflated upper tract carriage with lower tract infection. Serological methods (enzyme-linked immunosorbent assay [ELISA]) detected prior exposure rather than current infection [100,156,162], producing prevalence estimates that were biologically distinct from those generated by direct detection methods. Culture demonstrated high specificity but variable sensitivity (approximately 42–78%) compared with PCR [32,33], and studies using culture as the sole reference standard may have underestimated true prevalence. MALDI-TOF MS [62] and nanopore sequencing [63] offered novel approaches for species- and strain-level identification but remained limited to laboratory settings. Owing to the scarcity of standardised economic outcome data across studies, a formal cost-effectiveness comparison of diagnostic approaches was not undertaken in the current study. However, practical considerations such as infrastructure requirements, turnaround time, and field deployability are likely to influence real-world diagnostic selection. Among the newer diagnostic approaches evaluated in this review, loop-mediated isothermal amplification presently appears the most realistic candidate for near-term field or near-farm deployment because it combines rapid turnaround with lower technical complexity than sequencing-based or mass-spectrometry-based platforms [56,92,126].

These methodological differences meant that prevalence estimates could not be meaningfully pooled across studies, and direct comparison of reported detection rates was confounded by assay, sample type, and population differences. QUADAS-2 assessment [45] identified applicability concerns for reference standards in approximately one-third of diagnostic studies, primarily owing to this variability.

The inconsistent efficacy observed across vaccine platforms was explicable through defined biological mechanisms, and the pattern of platform-dependent outcomes provided indirect evidence for hypotheses about what constitutes protective immunity against a pathobiont.

Whole-cell bacterins, the most widely deployed platform, performed poorly in two randomised controlled trials, with no significant reduction in pneumonia incidence [30,31] and significantly increased otitis media in one trial [31]. Several features may explain this failure. Mycoplasmas lack a cell wall, eliminating the classical pathogen-associated molecular patterns (lipopolysaccharide, peptidoglycan) that serve as natural adjuvants in conventional bacterial vaccines [19,26]. While mycoplasmal lipoproteins activate TLR2, the resulting signalling cascade skews responses toward T-helper 2 (Th2) phenotypes characterised by interleukin (IL)-4, IL-5, and IL-13 production, rather than the Th1 responses (interferon-γ, tumour necrosis factor-α) associated with clearance of intracellular pathogens [86,224]. Aluminium-based adjuvants, commonly used in veterinary bacterins, reinforce rather than correct this Th2 skewing [90]. Furthermore, formalin inactivation during bacterin manufacture may alter conformational epitopes, reducing the fidelity of the immune response to native surface structures expressed during infection [29]. High-frequency antigenic variation through variable surface proteins (Vsps), switching at rates of 10^−2^ to 10^−4^ per generation [24,25], generated within-host phenotypic diversity that outpaced antibody-mediated selection, even with autogenous bacterins prepared from herd-specific isolates.

Intracellular survival within non-professional phagocytes (epithelial cells, monocytes) creates sanctuary sites inaccessible to extracellular antibodies [166,198]. Clearance of intracellular pathogens typically requires cytotoxic T lymphocyte (CTL) responses that recognise and destroy infected host cells presenting mycoplasma antigens on major histocompatibility complex (MHC) class I molecules [3,29]. However, killed whole-cell antigens delivered parenterally are processed predominantly through MHC class II pathways, inducing Th2-biased antibody responses while poorly stimulating the MHC class I presentation required for CTL induction [125,217]. Active immunomodulation subverts both natural and vaccine-induced immunity. Mycoplasmas possess many of these mechanisms, including arginine depletion suppressing lymphocyte proliferation, apoptosis induction in immune effector cells, regulatory T-cell expansion, and interleukin-10 (IL-10) production, dampening inflammatory responses [27,28,78]. Biofilm-mediated persistence at mucosal surfaces establishes structured communities resistant to immune effectors and antimicrobials [21]. Finally, mucosal immune compartmentalisation means that parenteral vaccination inadequately stimulates secretory immunoglobulin A (IgA) at the respiratory surfaces where infection is established [67,111]. The transudation of serum immunoglobulin G (IgG) onto mucosal surfaces is limited compared with actively transported secretory IgA, which is the principal antibody isotype mediating the mucosal immunity [29].

These considerations might explain why alternative platforms showed more promising results. Intranasal modified-live delivery stimulated mucosal-associated lymphoid tissue and induced respiratory IgA responses [67], addressing the compartmentalisation limitation of parenteral approaches. Subunit vaccines targeting specific virulence factors direct immunity toward pathogenicity mechanisms rather than commensal surface antigens, offering a rationale for disease prevention without elimination of carriage. Candidate subunit antigens investigated to date include elongation factor Tu and heat shock protein 70 [136], fibronectin-binding adhesin P27 [77], and plasminogen-binding fructose-1,6-bisphosphate aldolase [101]. Saponin-based adjuvant formulations stimulated both Th1 and Th2 responses, partially correcting the Th2 skewing inherent to mycoplasmal antigen presentation [88,90]. Combined *M. bovis*–BoHV-1 approaches demonstrated broad protection in experimental studies [109,232].

The risk of vaccine-enhanced disease further constrained development. Certain lipoprotein-based subunit candidates have caused exacerbated pathology, with immune complex deposition in pulmonary vasculature and type III hypersensitivity reactions reported following challenge [237]. Analogous observations in the related contagious bovine pleuropneumonia system, where live vaccine formulations produced variable and sometimes adverse outcomes [40,41], further underscore this risk. Critically, the positive signals for virulence-factor and mucosal platforms derive predominantly from controlled challenge models with standardised inocula and short follow-up periods. Evidence from field trials under natural exposure conditions remains sparse, limiting extrapolation to commercial production settings. This phenomenon underscores that correlates of protection remain incompletely defined, as antibody responses that appear protective in vitro may prove harmful in vivo [29,125,217].

Several limitations constrained the certainty and generalisability of these vaccine conclusions. Only 15 studies met the inclusion criteria for controlled vaccine evaluation. The RoB 2 assessment [43] identified some concerns regarding blinding in five and a high risk of bias for outcome measurement in two. The majority employed experimental challenge models rather than natural field exposure, limiting ecological validity. Heterogeneity in challenge strains, inoculation routes, endpoints (clinical scores, lung lesion scores, colonisation, or immune markers), case definitions, and follow-up duration precluded quantitative synthesis. Formal assessment of publication bias was not feasible owing to the heterogeneity of study designs, outcomes, and species across the four evidence domains. However, the predominance of studies reporting positive or significant findings, particularly for novel diagnostic assays and experimental vaccines, suggests that reporting bias may inflate apparent performance. Sensitivity analyses were not conducted because the heterogeneity precluded meta-analysis and meaningful sensitivity testing of pooled estimates. No field efficacy trials of virulence-factor vaccines or mucosal delivery platforms have been completed, meaning that translation to field conditions remains unvalidated. Field factors of importance include diverse pathogen strains, variable host immunity, and co-infections with viral (BHV-1, BVDV, BRSV) and bacterial (e.g., *M. haemolytica*, *P. multocida*) pathogens. Sterilising immunity, complete prevention of colonisation, is neither achievable nor biologically appropriate for an organism colonising a substantial proportion of healthy cattle [1,2,3].

The continuum framework [4,5] has several practical implications that differ from conventional single-pathogen approaches. Diagnostic interpretation must be species-aware and context-integrated. A positive detection of *M. bovirhinis* or *M. arginini* should not inform treatment decisions [7,8], whereas *M. bovis* detection requires integration with lesion localisation, quantitative burden, clinical syndrome, and epidemiological context before causal attribution is warranted [5,74]. For practical decision-making, features increasing confidence in *M. bovis* causation include lower respiratory tract sampling [35], high quantitative load [143], concurrent clinical signs, and absence of alternative sufficient causes [236].

Vaccine development should prioritise *M. bovis*-specific virulence factors with mucosal delivery, targeting pathogenicity mechanisms, rather than commensal surface antigens [67,77,136]. For vaccine development, virulence factors such as the fibronectin-binding adhesin *P27* [77], plasminogen-binding fructose-1,6-bisphosphate aldolase [101], and the conserved antigens elongation factor Tu and heat shock protein 70 can be considered [136]. Realistic endpoints should target disease mitigation and productivity improvement rather than sterilising immunity [29,41]. Careful safety evaluation is mandated, given the risk of vaccine-enhanced disease [40,41].

The New Zealand *M. bovis* eradication programme [132] demonstrated that integrated control, combining enhanced surveillance, movement restrictions, test-and-cull, and biosecurity measures, can achieve meaningful impact even without effective vaccines. In endemic settings where eradication is not feasible, integrated control combining syndrome-aligned diagnostics, targeted vaccination, biosecurity, and antimicrobial stewardship was more consistently supported than any single intervention [132,163]. The evidence base for integrated approaches was predominantly observational. The relative contributions of individual components have not been disentangled through controlled study designs. The available evidence did not support antimicrobial resistance acquisition as the primary determinant of the commensal-to-pathogen transition; rather, the transition was more consistently associated with virulence-associated traits, including antigenic variation, intracellular persistence, biofilm formation, and active immunomodulation, together with environmental co-factors such as viral co-infection and management-related stress [13,16,21,25,27,166,198]. Antimicrobial resistance could be interpreted as a factor that may exacerbate persistence and therapeutic failure once a clinically relevant infection is established.

On the basis of the gaps identified in this review, several research priorities emerge. Dedicated studies of non-*M. bovis* bovine respiratory mycoplasmas, particularly *M. dispar* and *M. arginini*, are needed to validate continuum classifications through controlled challenge designs, isolating species-specific pathogenic contributions. Multiplex diagnostic approaches [113,155] should become standard practice, accompanied by species-specific clinical methods that guide interpretation according to continuum position. Tissue localisation studies employing multiplex immunohistochemistry or fluorescent in situ hybridisation [22] should co-localise multiple pathogens within lesions, distinguishing organisms at lesion margins from those in necrotic centres. Standardised case definitions integrating clinical syndrome, multiplex pathogen profiles with quantitative loads, host response markers [83,111], and epidemiological context should be developed and validated. Randomised controlled trials of *M. bovis* virulence-factor vaccines [136] with disease endpoints, not colonisation alone, are needed, with careful safety evaluation given vaccine-enhanced disease risk [40]. Mucosal delivery should be evaluated with longer follow-up and transmission endpoints, comparing intranasal, aerosol, and heterologous prime–boost strategies [67]. Longitudinal cohorts tracking the commensal-to-pathogen transition with detailed risk factor assessment, multiplex pathogen monitoring, and host response profiling [11,12] would strengthen evidence for risk stratification. Economic evaluation of integrated control, quantifying the relative contributions of diagnostics, vaccination, biosecurity, and management, is needed for both eradication (*M. bovis*) [132] and endemic contexts.

## 5. Conclusions

Bovine respiratory mycoplasma species occupied distinct positions along a commensal–pathogen continuum, with direct implications for vaccine design, diagnostic interpretation, and disease control. *M. bovirhinis* and *M. arginini* behaved as commensals, *M. dispar* as an opportunist, and *M. bovis* as a pathobiont whose progression from carriage to disease was context-dependent. Whole-cell bacterins provided inconsistent protection, whereas virulence-factor and mucosal delivery vaccines produced more favourable outcomes, although field evidence remains limited. Detection of mycoplasma DNA or viable organisms alone was insufficient to infer causation; causal attribution required species identity, sampling depth, quantitative burden, tissue localisation, polymicrobial context, and clinical syndrome to be considered together. Integrated control combining syndrome-aligned diagnostics, biosecurity, targeted vaccination, and antimicrobial stewardship was the approach most consistently supported by the evidence. Priority research areas include non-*M. bovis* species, continuum-informed multiplex diagnostic methods, randomised controlled trials of vaccines with disease-based endpoints, and longitudinal studies of the commensal-to-pathogen transition.

## Figures and Tables

**Table 1:** Inclusion and exclusion criteria for study eligibility, organised by PICOS element.

Element	Inclusion Criteria	Exclusion Criteria ^a^
Population	Domestic cattle (Bos taurus, Bos indicus, and crosses) with mycoplasma infection, carriage, vaccination, or diagnostics within a bovine respiratory disease context.	Studies exclusively addressing non-bovine ruminants, non-ruminant species, or wild bovids without concurrent domestic cattle data; no mention of Mycoplasma, Mollicutes, or related genera; studies primarily addressing M. mycoides subsp. mycoides (CBPP); studies addressing exclusively non-respiratory syndromes (mastitis, arthritis, otitis, genital infections, contagious agalactia).
Intervention/Exposure	(a) Vaccination or immunisation against respiratory mycoplasmas (bacterins, live-attenuated, subunit, autogenous, mucosal delivery); (b) diagnostic methods (DTA studies reporting Se and/or Sp, or method-comparison studies); (c) carriage dynamics and prevalence; (d) pathogenesis and immune response.	Studies reporting solely in vitro antimicrobial susceptibility testing without associated clinical outcomes; assay development without validation against clinical or field samples.
Comparator	Vaccine studies: mandatory comparator (placebo, saline, adjuvant-only, or unvaccinated controls). Diagnostic studies: defined reference standard (culture, validated PCR, or composite reference). Carriage and pathogenesis studies: no comparator required.	Vaccine studies lacking randomised group allocation and a defined comparator (applied at full-text stage).
Outcomes	At least one quantifiable outcome: mortality or morbidity; clinical disease scores; lung lesion scores; colonisation, shedding, or transmission; immune responses; diagnostic performance (Se, Sp, PPV, NPV, AUC); prevalence or incidence with denominators; production parameters; effect estimates with precision.	—
Study design	RCTs and randomised experimental challenge studies (vaccine); DTA and method-comparison studies (diagnostic); cross-sectional, longitudinal, abattoir, and surveillance studies (carriage); experimental challenge and in vitro studies (pathogenesis).	Systematic reviews, narrative reviews, meta-analyses, scoping reviews, editorials, letters, conference abstracts, book chapters, errata, corrigenda, and retracted articles.

^a^ Exclusion criteria were applied hierarchically; any single criterion was sufficient for exclusion. Criterion E8 (vaccine study design) was applied at the full-text stage. AUC, area under the curve; CBPP, contagious bovine pleuropneumonia; DTA, diagnostic test accuracy; NPV, negative predictive value; PCR, polymerase chain reaction; PICOS, Population, Intervention, Comparator, Outcome, Study design; PPV, positive predictive value; RCT, randomised controlled trial; Se, sensitivity; Sp, specificity.

**Table 2:** Search strategy concept blocks and terms.

Concept Block	Search Terms
Block 1: Taxonomic identifiers	Mycoplasma*; Mycoplasmopsis*; Metamycoplasma*; Mesomycoplasma* ^a^
Block 2: Host species	ruminant*; cattle; bovine; cow; cows; calf; calves; sheep; ovine; goat*; caprine; buffalo*
Block 3: Intervention, diagnostic, and outcome terms	diagnos*; detection; misdiagnos*; attribution; causation; sensitivity; specificity; “false positive”; “false negative”; carriage; colonis*; coloniz*; vaccin*; immunization; immunisation; immunize*; immunise*; bacterin*; “vaccine efficacy”; “vaccine effectiveness”; failure; limitation*; underperform*; “non-sterilizing”; “nonsterilizing”; “protective immunity”; correlat* AND protect*; “immune response”; immunogen*; “immune evasion”; “immune escape”; “antigenic variation”; antigenic; immunopatholog*; biofilm*; persist*
Combination logic ^b^	Block 1 AND Block 2 AND Block 3; NOT (humans[MeSH] NOT animals[MeSH]) for PubMed; NOT TITLE-ABS-KEY(human*) for Scopus
Database-specific adaptations	PubMed: [Title/Abstract] field tags with MeSH exclusion filter. Web of Science: TS = topic search. Scopus: TITLE-ABS-KEY combined field search. Wildcards (*) and proximity operators adapted per database syntax.

^a^ Asterisks (*) denote truncation. Terms within each block were combined with OR. ^b^ Blocks were combined with AND; exclusion filters were applied per database syntax.

**Table 3:** Characteristics of included studies by domain, publication period, and geographic region (*n* = 212).

Characteristic	Carriage	Diagnostic	Pathogenesis	Vaccine
Total studies, n (%) ^a^	73 (34.4)	71 (33.5)	53 (25.0)	15 (7.1)
Publication period				
Pre-2000	8	8	4	3
2000–2009	4	8	5	2
2010–2019	28	32	22	6
2020–2026	33	23	22	4
Geographic region				
Europe	26	24	17	5
Americas	22	20	15	5
Asia	13	14	12	3
Oceania	6	5	3	1
South America	4	5	3	1
Africa	2	3	3	0

^a^ Values are study counts; percentages of total included studies (*n* = 212) are shown for domain totals. Studies may appear in more than one domain; domain totals, therefore, sum to more than 212.

**Table 4:** Summary of risk-of-bias assessments by study domain and assessment tool.

Domain	Tool	Low Risk ^a^	Some Concerns	High Risk
Vaccine (*n* = 15)	RoB 2	8 (53%)	5 (33%)	2 (13%)
Diagnostic (*n* = 71)	QUADAS-2	45 (63%)	18 (25%)	8 (11%)
Carriage (*n* = 73)	NOS	38 (52%)	24 (33%)	11 (15%)
Pathogenesis (*n* = 53)	ROBINS-I/NOS	29 (55%)	16 (30%)	8 (15%)

^a^ Values are study counts with percentages of the domain total in parentheses. NOS, Newcastle–Ottawa Scale; QUADAS-2, quality assessment of diagnostic accuracy studies version 2; RoB 2, Cochrane Risk of Bias tool version 2; ROBINS-I, risk of bias in non-randomised studies of interventions.

**Table 5:** *Mycoplasma* species distribution across study domains (*n* = 212).

Species	Carriage ^a^	Diagnostic ^a^	Pathogenesis ^a^	Vaccine ^a^	Continuum ^c^
*M. alkalescens*	0	1	0	0	Occasional respiratory isolate
*M. arginini*	3	1	0	0	True commensal
*M. bovirhinis*	14	3	2	0	True commensal
*M. bovis* ^b^	64	66	47	15	Pathobiont
*M. canadense*	1	0	0	0	Occasional respiratory isolate
*M. dispar*	12	5	4	1	Commensal/opportunist

^a^ Within each domain, multi-species studies are counted under each species reported; column totals may therefore exceed the domain study count. ^b^ For *M. bovis*, the row total (192) is lower than the overall mention count reported in the text (199/212) because seven studies detected *M. bovis* incidentally but were classified under another species for their primary domain analysis. ^c^ Continuum position assigned based on evidence synthesis (see Section 3.4). Species names follow revised Mollicutes taxonomy; traditional *Mycoplasma* abbreviations are retained for readability.

**Table 6:** Summary of vaccine efficacy by platform, delivery route, and primary endpoint (*n* = 15 studies).

Platform	n	Route	Primary Endpoint	Effect Direction ^a^
Whole-cell bacterin	4	Parenteral (SC/IM)	Pneumonia incidence	Inconsistent (2○, 1+, 1−)
Live attenuated	2	Parenteral/intranasal	Clinical disease	Positive (2+)
Subunit/recombinant	3	Parenteral (IM)	Challenge protection	Partially positive (2+, 1○)
Saponin-adjuvanted	3	Parenteral (SC)	Immune response	Positive (3+)
Intranasal modified-live	1	Intranasal	Mucosal IgA	Positive (1+)
Combined (*M. bovis*–BoHV-1) ^b^	2	Parenteral/intranasal	Broad protection	Positive (2+)

^a^ Effect direction: +, statistically significant benefit; ○, no significant effect; −, statistically significant harm. ^b^ Combined *M. bovis*–BoHV-1 multivalent vaccine formulations. BoHV-1, bovine herpesvirus 1; IgA, immunoglobulin A; IM, intramuscular; SC, subcutaneous.

## Data Availability

All data generated or analysed during this study are included in this published article and its Supplementary Information Files.

## Supplementary Materials

The following supporting information can be downloaded at: https://www.mdpi.com/article/10.3390/ani16060960/s1. Supplementary Material S1: PRISMA 2020 Checklist; Supplementary Material S2: Complete Search Strategies; Supplementary Material S3: Data Extraction Template; Supplementary Material S4: GRADE Summary of Findings; Supplementary Material S5: Risk of Bias Assessments; Supplementary Material S6: Synthesis Without Meta-analysis (SWiM) reporting checklist; Figure S1: PRISMA 2020 Flow Diagram.
