# Supplementary material for: Bovine Respiratory Mycoplasmas and the Commensal–Pathogen Continuum: A Systematic Review of Vaccines and Diagnostic Approaches
Source: Animals (Basel). 2026 Mar 19;16(6):960. doi: 10.3390/ani16060960 (PMC13023341; doi:10.3390/ani16060960)
Supplement: Supplementary file 1 [file animals-16-00960-s001.zip › Figure_S1_PRISMA_Flow_Diagram.pdf]

Supplementary Figure S1: PRISMA 2020 Flow Diagram

IDENTIFICATION

Records identified through database searching (n = 6119) PubMed (n = 1562) | Scopus (n = 2461) | Web of Science (n = 2096)

↓

Records after duplicate removal (n = 3422) Duplicates removed (n = 2697)

SCREENING

↓

Records screened by title and abstract (n = 3422) κ = 0.780 (95% CI: 0.740–0.820) → Records excluded (n = 2575)

↓

Full-text articles assessed (n = 847) κ = 0.910 (95% CI: 0.870–0.950) → Full-text excluded (n = 635) • Wrong population (n = 201) • Reviews/meta-analyses (n = 112) • Insufficient methods (n = 108) • Antimicrobial susceptibility only (n = 67) • Duplicate data (n = 54) • Method development only (n = 38) • Non-English language (n = 32) • Other (n = 23)

INCLUDED

↓

Studies included in narrative synthesis (n = 212)

Carriage/ Prevalence (n = 73; 34.4%)	Diagnostic Performance (n = 71; 33.5%)	Pathogenesis/ Immune Evasion (n = 53; 25.0%)	Vaccine Efficacy (n = 15; 7.1%)
