# Supplementary material for: Bovine Respiratory Mycoplasmas and the Commensal–Pathogen Continuum: A Systematic Review of Vaccines and Diagnostic Approaches
Source: Animals (Basel). 2026 Mar 19;16(6):960. doi: 10.3390/ani16060960 (PMC13023341; doi:10.3390/ani16060960)
Supplement: Supplementary file 1 [file animals-16-00960-s001.zip › S1_PRISMA_2020_Checklist.pdf]

# Supplementary Material S1: PRISMA 2020 Checklist

| Section and topic | Item | # | Checklist item | Location |
|---|---|---|---|---|
| **TITLE** | Title | 1 | Identify the report as a systematic review. | Title page |
| **ABSTRACT** | Abstract | 2 | See the PRISMA 2020 for Abstracts checklist. | Abstract |
| **INTRODUCTION** | Rationale | 3 | Describe the rationale for the review in the context of existing knowledge. | Section 1 |
| **INTRODUCTION** | Objectives | 4 | Provide an explicit statement of the objective(s) or question(s) the review addresses. | Section 1, final paragraph |
| **METHODS** | Eligibility criteria | 5 | Specify the inclusion and exclusion criteria for the review and how studies were grouped for the syntheses. | Section 2.2; Table 1 |
| **METHODS** | Information sources | 6 | Specify all databases, registers, websites, organisations, reference lists and other sources searched or consulted to identify studies. | Section 2.3 |
| **METHODS** | Search strategy | 7 | Present the full search strategies for all databases, registers and websites, including any filters and limits used. | Section 2.3; Table 2; S2 |
| **METHODS** | Selection process | 8 | Specify the methods used to decide whether a study met the inclusion criteria of the review. | Section 2.4; S8 |
| **METHODS** | Data collection process | 9 | Specify the methods used to collect data from reports. | Section 2.5; S3 |
| **METHODS** | Data items | 10a | List and define all outcomes for which data were sought. | Section 2.5 |
| **METHODS** | Data items | 10b | List and define all other variables for which data were sought. | Section 2.5; S3 |
| **METHODS** | Study risk of bias assessment | 11 | Specify the methods used to assess risk of bias in the included studies. | Section 2.6; S6 |
| **METHODS** | Effect measures | 12 | Specify for each outcome the effect measure(s) used in the synthesis or presentation of results. | Section 2.7 |
| **METHODS** | Synthesis methods | 13a | Describe the processes used to decide which studies were eligible for each synthesis. | Section 2.7 |
| **METHODS** | Synthesis methods | 13b | Describe any methods required to prepare the data for presentation or synthesis. | Section 2.7 |
| **METHODS** | Synthesis methods | 13c | Describe any methods used to tabulate or visually display results of individual studies and syntheses. | Section 2.7; Tables 3–6 |
| **METHODS** | Synthesis methods | 13d | Describe any methods used to synthesise results and provide a rationale for the choice(s). | Section 2.7; S7 |
| **METHODS** | Synthesis methods | 13e | Describe any methods used to explore possible causes of heterogeneity among study results. | Section 2.7 |
| **METHODS** | Synthesis methods | 13f | Describe any sensitivity analyses conducted to assess robustness of the synthesised results. | Section 2.7; Section 4 |
| **METHODS** | Reporting bias assessment | 14 | Describe any methods used to assess risk of bias due to missing results in a synthesis. | Section 2.7; Section 4 |
| **METHODS** | Certainty assessment | 15 | Describe any methods used to assess certainty in the body of evidence for an outcome. | Section 2.8; S4 |
| **RESULTS** | Study selection | 16a | Describe the results of the search and the process for selecting studies. | Section 3.1; Figure S1 |
| **RESULTS** | Study selection | 16b | Cite studies that might appear to meet the inclusion criteria, but which were excluded, and explain why they were excluded. | Section 3.1 |

| | | | | |
|---|---|---|---|---|
| **RESULTS** | Study characteristics | 17 | Cite each included study and present its characteristics. | Sections 3.2–3.4; Tables 3–5; S1; S5 |
| **RESULTS** | Risk of bias in studies | 18 | Present assessments of risk of bias for each included study. | Section 3.3; Table 4; S6 |
| **RESULTS** | Results of individual studies | 19 | For all outcomes, present, for each study: summary statistics and an effect estimate and its precision. | Sections 3.5–3.8 |
| **RESULTS** | Results of syntheses | 20a | For each synthesis, briefly summarise the characteristics and risk of bias among contributing studies. | Sections 3.5–3.8 |
| **RESULTS** | Results of syntheses | 20b | Present results of all statistical syntheses conducted. | Not applicable (narrative synthesis; S7) |
| **RESULTS** | Results of syntheses | 20c | Present results of all investigations of possible causes of heterogeneity among study results. | Sections 3.5–3.8; Section 4 |
| **RESULTS** | Results of syntheses | 20d | Present results of all sensitivity analyses conducted to assess the robustness of the synthesised results. | Section 4 |
| **RESULTS** | Reporting biases | 21 | Present assessments of risk of bias due to missing results for each synthesis assessed. | Section 4 |
| **RESULTS** | Certainty of evidence | 22 | Present assessments of certainty in the body of evidence for each outcome assessed. | Section 3.3; Table 4; S4 |
| **DISCUSSION** | Discussion | 23a | Provide a general interpretation of the results in the context of other evidence. | Section 4 |
| **DISCUSSION** | Discussion | 23b | Discuss any limitations of the evidence included in the review. | Section 4 |
| **DISCUSSION** | Discussion | 23c | Discuss any limitations of the review processes used. | Section 4 |
| **DISCUSSION** | Discussion | 23d | Discuss implications of the results for practice, policy, and future research. | Section 4; Section 5 |
| **OTHER INFORMATION** | Registration and protocol | 24a | Provide registration information for the review. | Section 2.1 |
| **OTHER INFORMATION** | Registration and protocol | 24b | Indicate where the review protocol can be accessed. | Section 2.1 |
| **OTHER INFORMATION** | Registration and protocol | 24c | Describe and explain any amendments to information provided at registration or in the protocol. | Section 2.1 (no deviations) |
| **OTHER INFORMATION** | Support | 25 | Describe sources of financial or non-financial support for the review. | Funding statement |
| **OTHER INFORMATION** | Competing interests | 26 | Declare any competing interests of review authors. | Conflicts of Interest |
| **OTHER INFORMATION** | Availability of data, code and other materials | 27 | Report which of the following are publicly available and where they can be found. | Data Availability Statement; S1; S3; S5 |
