# Supplementary material for: Bovine Respiratory Mycoplasmas and the Commensal–Pathogen Continuum: A Systematic Review of Vaccines and Diagnostic Approaches
Source: Animals (Basel). 2026 Mar 19;16(6):960. doi: 10.3390/ani16060960 (PMC13023341; doi:10.3390/ani16060960)
Supplement: Supplementary file 1 [file animals-16-00960-s001.zip › S2_Complete_Search_Strategies.pdf]

Supplementary Material S2: Complete Search Strategies

1. PubMed/MEDLINE

Platform: NLM via PubMed. Fields: [Title/Abstract] with MeSH exclusion filter. Records: 1562.

```
((Mycoplasma*[Title/Abstract]
  OR Mycoplasmopsis*[Title/Abstract]
  OR Metamycoplasma*[Title/Abstract]
  OR Mesomycoplasma*[Title/Abstract])
AND
(ruminant*[Title/Abstract]
  OR cattle[Title/Abstract]
  OR bovine[Title/Abstract]
  OR cow[Title/Abstract]
  OR cows[Title/Abstract]
  OR calf[Title/Abstract]
  OR calves[Title/Abstract]
  OR sheep[Title/Abstract]
  OR ovine[Title/Abstract]
  OR goat*[Title/Abstract]
  OR caprine[Title/Abstract]
  OR buffalo*[Title/Abstract])
AND
(diagnos*[Title/Abstract]
  OR detection[Title/Abstract]
  OR misdiagnos*[Title/Abstract]
  OR attribution[Title/Abstract]
  OR causation[Title/Abstract]
  OR sensitivity[Title/Abstract]
  OR specificity[Title/Abstract]
  OR "false positive"[Title/Abstract]
  OR "false negative"[Title/Abstract]
  OR carriage[Title/Abstract]
  OR colonis*[Title/Abstract]
  OR coloniz*[Title/Abstract]
  OR vaccin*[Title/Abstract]
  OR immunization[Title/Abstract]
  OR immunisation[Title/Abstract]
  OR immunize*[Title/Abstract]
  OR immunise*[Title/Abstract]
  OR bacterin*[Title/Abstract]
  OR "vaccine efficacy"[Title/Abstract]
  OR "vaccine effectiveness"[Title/Abstract]
  OR failure[Title/Abstract]
  OR limitation*[Title/Abstract]
  OR underperform*[Title/Abstract]
  OR "non-sterilizing"[Title/Abstract]
  OR "nonsterilizing"[Title/Abstract]
  OR "protective immunity"[Title/Abstract]
  OR (correlat*[Title/Abstract] AND protect*[Title/Abstract])
  OR "immune response"[Title/Abstract]
  OR immunogen*[Title/Abstract]
  OR "immune evasion"[Title/Abstract]
  OR "immune escape"[Title/Abstract]
```

```
  OR "antigenic variation"[Title/Abstract]
  OR antigenic[Title/Abstract]
  OR immunopatholog*[Title/Abstract]
  OR biofilm*[Title/Abstract]
  OR persist*[Title/Abstract]))
NOT
(humans[MeSH Terms] NOT animals[MeSH Terms])
```

2. Web of Science Core Collection

Platform: Clarivate Analytics. Fields: TS= (Topic Search). Records: 2096.

```
TS=((Mycoplasma* OR Mycoplasmopsis*
  OR Metamycoplasma* OR Mesomycoplasma*)
AND
(ruminant* OR cattle OR bovine OR cow*
  OR calf OR calves OR sheep OR ovine
  OR goat* OR caprine OR buffalo*)
AND
(diagnos* OR detection OR attribution
  OR causation OR sensitivity OR specificity
  OR "false positive*" OR "false negative*"
  OR carriage OR colonis* OR coloniz*
  OR vaccin* OR immuni?ation OR bacterin*
  OR "vaccine efficacy" OR "vaccine effectiveness"
  OR failure OR limitation* OR underperform*
  OR "non-sterilizing" OR "protective immunity"
  OR "correlate* of protection"
  OR "immune response" OR "immune evasion"
  OR "antigenic variation" OR immunopatholog*
  OR biofilm*))
```

3. Scopus

Platform: Elsevier. Fields: TITLE-ABS-KEY. Records: 2461.

```
TITLE-ABS-KEY(
(Mycoplasma* OR Mycoplasmopsis*
  OR Metamycoplasma* OR Mesomycoplasma*)
AND
(ruminant* OR cattle OR bovine OR cow*
  OR calf OR calves OR sheep OR ovine
  OR goat* OR caprine OR buffalo*)
AND
(diagnos* OR detection OR attribution
  OR causation OR sensitivity OR specificity
  OR "false positive*" OR "false negative*"
  OR carriage OR colonis* OR coloniz*
  OR vaccin* OR immuni?ation OR bacterin*
  OR "vaccine efficacy" OR "vaccine effectiveness"
  OR failure OR limitation* OR underperform*
  OR "non-sterilizing" OR "protective immunity"
  OR "correlate* of protection"
  OR "immune response" OR "immune evasion"
  OR "antigenic variation" OR immunopatholog*
  OR biofilm*))
AND NOT TITLE-ABS-KEY(human*)
```
