# Supplementary material for: Bovine Respiratory Mycoplasmas and the Commensal–Pathogen Continuum: A Systematic Review of Vaccines and Diagnostic Approaches
Source: Animals (Basel). 2026 Mar 19;16(6):960. doi: 10.3390/ani16060960 (PMC13023341; doi:10.3390/ani16060960)
Supplement: Supplementary file 1 [file animals-16-00960-s001.zip › S3_Data_Extraction_Template.pdf]

Supplementary Material S3: Data Extraction Template

Developed in Microsoft Excel and piloted on 20 randomly selected studies. The template captured variables across eight domains as listed below. The complete Excel file is available from the corresponding author.

**Domain**	**Variable**	**Description / coding instructions**	**Applicable domains**
**(i) Study ID**	Authors	All authors listed	All
	Year	Publication year	All
	Journal	Journal name, volume, pages	All
	Country	Country where study was conducted	All
	DOI	Digital Object Identifier	All
	Funding	Funding bodies; potential conflicts of interest	All
**(ii) Design**	Design_type	RCT; non-randomised intervention; DTA; cross-sectional; cohort; case series; challenge	All
	Blinding	Double-blind; single-blind; open-label; not applicable	Vaccine, Diagnostic
	Randomisation	Method of randomisation or allocation	Vaccine
**(iii) Population**	Host_species	Bos taurus; Bos indicus; crosses	All
	Breed	Breed(s) studied	All
	Age_class	Neonatal; calf (<6 mo); yearling; adult; mixed	All
	Production_system	Dairy; beef feedlot; cow-calf; veal; mixed	All
	Sample_size	Number of animals enrolled	All
	Health_status	Clinically healthy; diseased; mixed	All
**(iv) Clinical context**	Syndrome	Pneumonia; BRDC; otitis; arthritis; mastitis; surveillance	All
	Case_definition	Definition used for disease classification	All
	Outcome_measures	Primary and secondary outcomes reported	All
**(v) Target organism**	Mycoplasma_species	Species identified (legacy + revised nomenclature)	All
	ID_method	Culture; PCR; sequencing; MALDI-TOF; serology	All
	Strain_typing	MLST; MLVA; WGS; not performed	All
	Continuum_position	True commensal; commensal/opportunist; pathobiont; occasional isolate	All

**(vi) Co-pathogen**	Co_infection_assessed	Yes/No	All
	Co_pathogens	Organisms detected and methods used	All
**(vii) Vaccine**	Vaccine_type	Whole-cell bacterin; live attenuated; subunit; DNA; recombinant vector; autogenous	Vaccine
	Antigen_target	Specific antigen(s) or whole organism	Vaccine
	Adjuvant	Type and concentration	Vaccine
	Route	SC; IM; IN; intramammary; combined	Vaccine
	Dose_schedule	Number of doses, interval, timing relative to challenge/exposure	Vaccine
	Comparator	Placebo; saline; adjuvant-only; unvaccinated; alternative vaccine	Vaccine
	Effect_direction	+, statistically significant benefit; ○, null; −, harm	Vaccine
	Effect_size	RR, RRR, PE with 95% CI where reported	Vaccine
	Follow_up	Duration of follow-up post-vaccination	Vaccine
**(viii) Diagnostic**	Assay_type	Culture; conventional PCR; qPCR; ELISA; CFT; IHC; ISH; LAMP; MALDI-TOF; WGS	Diagnostic
	Target_gene	Gene target (e.g. oppD/F, uvrC, 16S rRNA, gyrB)	Diagnostic
	Ct_threshold	Cycle threshold or Cq for qPCR positivity	Diagnostic
	Reference_standard	Gold standard used for comparison	Diagnostic
	Sensitivity	Se with 95% CI if reported	Diagnostic
	Specificity	Sp with 95% CI if reported	Diagnostic
	Tissue_localisation	Yes/No; if yes, method (IHC, ISH, histopathology)	Diagnostic
	Sampling_site	Superficial nasal; deep NP; BAL; lung tissue; joint fluid; milk; blood	Diagnostic
**Quality**	RoB_tool	RoB 2; ROBINS-I; QUADAS-2; NOS	All
	Overall_RoB	Low; some concerns; high; serious; critical	All
	GRADE_certainty	High; moderate; low; very low	All

BAL, bronchoalveolar lavage; BRDC, bovine respiratory disease complex; CFT, complement fixation test; CI, confidence interval; DTA, diagnostic test accuracy; IHC, immunohistochemistry; IM, intramuscular; IN, intranasal; ISH, in situ hybridisation; LAMP, loop-mediated isothermal amplification; MALDI-TOF, matrix-assisted laser desorption/ionisation time-of-flight; MLST, multi-locus sequence typing; MLVA, multi-locus variable-number tandem-repeat analysis; NOS, Newcastle–Ottawa Scale; NP, nasopharyngeal; PCR, polymerase chain reaction; PE, protective efficacy; qPCR, quantitative PCR; RCT, randomised controlled trial; RoB, risk of bias; RR, relative risk; RRR, relative risk reduction; SC, subcutaneous; Se, sensitivity; Sp, specificity; WGS, whole-genome sequencing.
