# Supplementary material for: Bovine Respiratory Mycoplasmas and the Commensal–Pathogen Continuum: A Systematic Review of Vaccines and Diagnostic Approaches
Source: Animals (Basel). 2026 Mar 19;16(6):960. doi: 10.3390/ani16060960 (PMC13023341; doi:10.3390/ani16060960)
Supplement: Supplementary file 1 [file animals-16-00960-s001.zip › S4_GRADE_Summary_of_Findings.pdf]

Supplementary Material S4: GRADE Summary of Findings Tables

Table S4.1. Vaccine efficacy by platform (n = 15 controlled studies)

Platform	n	Route	Primary endpoint	Effect direction	Key findings (from manuscript)	GRADE
Whole-cell bacterin	4	Parenteral (SC/IM)	Pneumonia incidence	Inconsistent (2○, 1+, 1−)	Two RCTs: no significant reduction in pneumonia [151,192]; one documented significantly increased otitis media in vaccinated calves [192]	**Very Low**
Live attenuated	2	Parenteral/intranasal	Clinical disease	Positive (2+)		**Low**
Subunit/recombinant	3	Parenteral (IM)	Challenge protection	Partially positive (2+, 1○)	EF-Tu and Hsp70: partial protection in experimental challenge [125]	**Low**
Saponin-adjuvanted	3	Parenteral (SC)	Immune response	Positive (3+)	Stimulated both Th1 and Th2 responses [77,79]	**Low**
Intranasal modified-live	1	Intranasal	Mucosal IgA	Positive (1+)	Promise for inducing mucosal immunity [50]	**Very Low**
Combined (M. bovis–BoHV-1)	2	Parenteral/intranasal	Broad protection	Positive (2+)	Broad protection in experimental studies [98,233]	**Low**

BoHV-1, bovine herpesvirus 1; EF-Tu, elongation factor thermo-unstable; Hsp70, heat shock protein 70; IgA, immunoglobulin A; IM, intramuscular; RCT, randomised controlled trial; SC, subcutaneous; Th1/Th2, T helper type 1/2.

Table S4.2. Diagnostic test performance by method (n = 71 studies)

Data from manuscript Section 3.6 and Discussion. Specific sensitivity and specificity values with 95% confidence intervals were not reported in the manuscript due to heterogeneity in sampling site, disease spectrum, index test design, threshold definition, and reference standard (Section 2.7). Findings are presented narratively as described in the manuscript.

Method	Target	Key findings (from Sections 3.6 and 4)	Key limitations	GRADE
Culture	M. bovis	High specificity but variable sensitivity (approximately 42–78%) compared with PCR [55,67]. Sensitivity lowest with recent antimicrobial treatment.	Requires 7–14 days; cold-chain dependent; cannot detect non-viable organisms.	**Low**
Quantitative PCR	M. bovis	High sensitivity and specificity [102,132,168]. No universally validated Cq threshold distinguishing carriage from infection; Cq ≤21.3 proposed [132] but population-dependent.	Detection does not equate to causation; positivity in healthy carriers complicates interpretation.	**Low**
LAMP	M. bovis	Promise for rapid, field-deployable detection [37,81,115,167]. Most realistic candidate for near-term field deployment.	Limited validation studies; not yet widely standardised.	**Very Low**
Multiplex PCR	M. bovis + co-pathogens	Simultaneous detection of multiple respiratory pathogens including M. bovis with M. dispar and M. bovirhinis [54,144].	Applicability varies with panel composition; species-specific interpretation needed.	**Very Low**
Serology (ELISA)	M. bovis	Detected immune responses (prior exposure) rather than current infection [89,145].	Delayed seroconversion; herd-level vs individual animal performance differs.	**Low**
MALDI-TOF MS	Mycoplasma spp.	Species- and strain-level identification [43].	Laboratory-based only; limited field applicability.	**Very Low**
Nanopore sequencing	Mycoplasma spp.	Species- and strain-level identification [44].	Laboratory-based; limited clinical validation.	**Very Low**
Deep NP swabs vs BAL	M. bovis	Deep NP swabs showed approximately 87% agreement with BAL vs only 52% for superficial nasal swabs [73,211,216].	Sampling depth significantly affects diagnostic inference; studies using superficial sampling conflate upper tract carriage with lower tract infection.	**Low**

BAL, bronchoalveolar lavage; Cq, quantification cycle; ELISA, enzyme-linked immunosorbent assay; LAMP, loop-mediated isothermal amplification; MALDI-TOF MS, matrix-assisted laser desorption/ionisation time-of-flight mass spectrometry; NP, nasopharyngeal; PCR, polymerase chain reaction.
