# Supplementary material for: Bovine Respiratory Mycoplasmas and the Commensal–Pathogen Continuum: A Systematic Review of Vaccines and Diagnostic Approaches
Source: Animals (Basel). 2026 Mar 19;16(6):960. doi: 10.3390/ani16060960 (PMC13023341; doi:10.3390/ani16060960)
Supplement: Supplementary file 1 [file animals-16-00960-s001.zip › S5_Risk_of_Bias_Assessments.pdf]

Supplementary Material S5: Risk of Bias Assessments

Table S5.1. RoB 2: Vaccine domain (n = 15)

Overall judgement	n (%)	Key issues (from Section 3.3)
**Low risk**	8 (53%)	Adequate randomisation, blinding, complete follow-up, pre-specified endpoints.
**Some concerns**	5 (33%)	Primarily inadequate blinding of outcome assessors; open-label designs with subjective clinical scoring.
**High risk**	2 (13%)	High risk of bias for outcome measurement: non-blinded assessment of subjective endpoints.

Table S5.2. QUADAS-2: Diagnostic domain (n = 71)

Overall judgement	n (%)	Key issues (from Section 3.3)
**Low risk**	45 (63%)	Low risk for patient selection and index test conduct in the majority.
**Some concerns**	18 (25%)	Applicability concerns for reference standards in approximately one-third, primarily owing to variability in reference standard selection.
**High risk**	8 (11%)	Reference standard variability; incorporation bias.

Table S5.3. NOS: Carriage/prevalence domain (n = 73)

Quality category	n (%)	Key issues
**Good (low risk)**	38 (52%)	Representative sampling frames; adequate response rates; validated detection methods.
**Fair (some concerns)**	24 (33%)	Moderate sample sizes; some limitations in ascertainment or non-response documentation.
**Poor (high risk)**	11 (15%)	Convenience samples; unclear case definitions; inadequate response rate documentation.

Table S5.4. ROBINS-I/NOS: Pathogenesis and immune evasion domain (n = 53)

Risk category	n (%)	Key issues
**Low risk**	29 (55%)	Well-controlled experimental challenge studies; adequate reporting of methods and outcomes.
**Some concerns/moderate**	16 (30%)	Incomplete adjustment for co-infections; small sample sizes in in vitro studies; partial blinding.
**High/serious risk**	8 (15%)	Major confounding without adjustment; selective outcome reporting; inadequate control groups.
