# Supplementary material for: Bovine Respiratory Mycoplasmas and the Commensal–Pathogen Continuum: A Systematic Review of Vaccines and Diagnostic Approaches
Source: Animals (Basel). 2026 Mar 19;16(6):960. doi: 10.3390/ani16060960 (PMC13023341; doi:10.3390/ani16060960)
Supplement: Supplementary file 1 [file animals-16-00960-s001.zip › S6_SWiM_Checklist.pdf]

Supplementary Material S6: Synthesis Without Meta-analysis (SWiM) Reporting Checklist

Item	SWiM reporting item	How addressed	Location
**1**	**Grouping studies for synthesis**	Four predefined thematic domains: (i) commensal carriage and species-specific continuum classification; (ii) vaccine efficacy; (iii) diagnostic performance; (iv) pathogenesis and immune evasion.	Section 2.7
**2**	**Standardised metric and transformation methods**	Effect direction: positive (+), null (○), or negative (−). Effect sizes with 95% CIs where available. Pooled Se/Sp not undertaken due to heterogeneity.	Section 2.7
**3**	**Synthesis methods**	Thematic narrative synthesis following SWiM [2]. Effect-direction analysis (structured vote counting). No quantitative meta-analysis.	Section 2.7
**4**	**Criteria to prioritise results**	Effect-direction analysis summarised findings. Studies at high risk of bias assigned less weight. GRADE certainty informed interpretation.	Sections 2.7–2.8
**5**	**Methods to investigate heterogeneity**	Sources examined by vaccine platform, host species, study design, geographic region. Results presented by species and platform.	Sections 3.5–3.8
**6**	**Certainty of evidence assessment**	GRADE approach [10]. Summary of Findings tables in S4. Adapted for diagnostic accuracy.	Section 2.8; S4
**7**	**Description of available data**	All extracted data in S5. Included studies in S1. Study characteristics in Tables 3–6.	Tables 3–6; S1; S5
**8**	**Synthesis methods used**	Narrative synthesis with effect-direction analysis. Formal publication bias assessment not feasible. Sensitivity analyses not conducted.	Section 2.7; Section 4
**9**	**Limitations of the synthesis**	Meta-analysis precluded by heterogeneity; vote counting gives equal weight; publication bias not formally assessable; concentration on M. bovis (93.9%).	Section 4
